# Hémangiome intestinal: une cause inhabituelle d'hémorragie digestive inexpliquée, rapport d'un cas

**DOI:** 10.11604/pamj.2014.19.64.4665

**Published:** 2014-09-24

**Authors:** Ghizlane Kharrasse, Hanane Hadjkacem, Rachid Azouguagh, Mehdi Soufi, Omar El Malki, Moulay Ezzahy Ismaili, Raouf Mohcine, Lahcen Ifrine, Abdelkader Belkouchi

**Affiliations:** 1 Service de Gastroentérologie, Hôpital El Farabi, Université Mohammed I, Oujda, Maroc; 2 Service de Radiologie, Hôpital El Farabi Université Mohammed I, Oujda, Maroc; 3 Service de Chirurgie A, CHU Avicenne, Rabat, Maroc

**Keywords:** Hémangiome, intestin, vidéo capsule, hémorragie inexpliquée, Hemangioma, intestin, video capsule, unexplained gastrointestinal bleeding

## Abstract

Les hémangiomes de l'intestin grêle (HG) sont des tumeurs bénignes et rares du tube digestif, parfois responsables d'hémorragie digestive. Le diagnostic positif de ces lésions est souvent difficile mais rendu plus aisé grâce à l'avènement de la vidéo capsule endoscopique (VCE), le traitement est essentiellement chirurgical. Nous rapportons une nouvelle observation d'un hémangiome caverneux du jéjunum révélé par une hémorragie digestive récidivante et diagnostiqué par VCE et nous discutons l'apport de cet examen dans la prise en charge de l'hémorragie digestive inexpliquée tout en la comparant aux autres explorations actuellement disponibles.

## Introduction

Les angiomes sont des tumeurs vasculaires fréquemment localisées au niveau de la peau. Les localisations digestives et notamment intestinales sont rares [1]. Le diagnostic de ces tumeurs peut s'avérer difficile surtout dans les localisations grêliques. Nous rapportons une nouvelle observation d'un hémangiome jéjunal révélé par une hémorragie digestive et nous insistons sur l'intérêt de la VCE dans le diagnostic étiologique en cas d'hémorragie digestive inexpliquée.

## Patient et observation

Il s'agit d'un patient âgé de 40 ans opéré en 2006 pour ulcère bulbaire perforé compliqué de péritonite, le malade a été hospitalisé dans notre unité pour prise en charge diagnostique et thérapeutique de méléna; en fait le patient a été aussi hospitalisé en 2008 pour un épisode hémorragique similaire, à cette époque il avait bénéficié d'une gastroscopie et d'une coloscopie ainsi qu'un angiocanner abdominal n'ayant retrouvé aucune anomalie, le malade est sorti sous traitement martial et il a été perdu de vue. Il a été ré admis aux urgences en 2010 pour méléna puis adressé à notre unité pour bilan étiologique.

**Figure 1:**
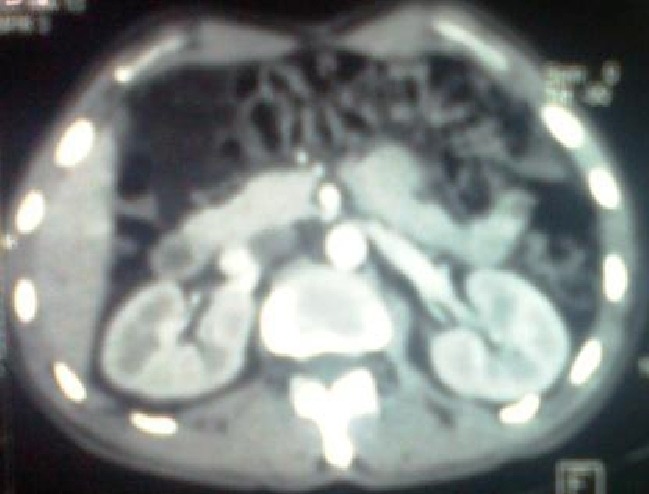
Coupe scannographique sans particularités (pas d’épaississement de la paroi intestinale et pas d'adénopathies abdominales)

**Figure 2:**
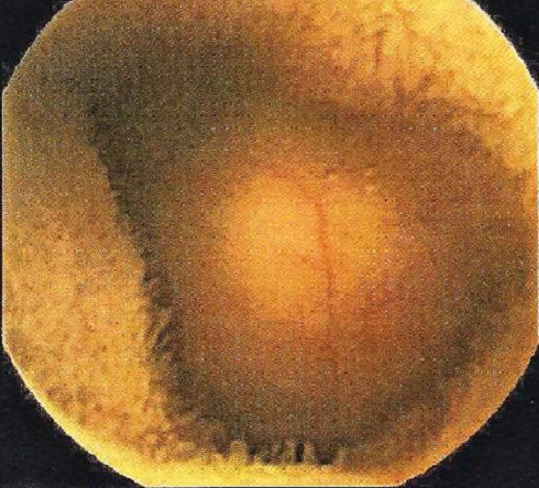
Tumeur sous muqueuse retardant le passage de la VCE

**Figure 3:**
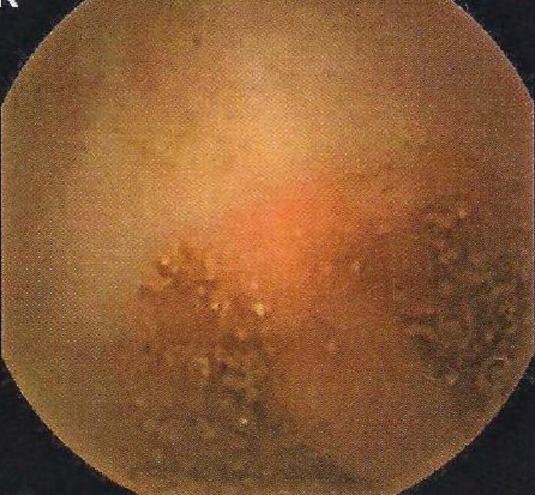
Stigmates de saignement récent au niveau iléal

**Figure 4:**
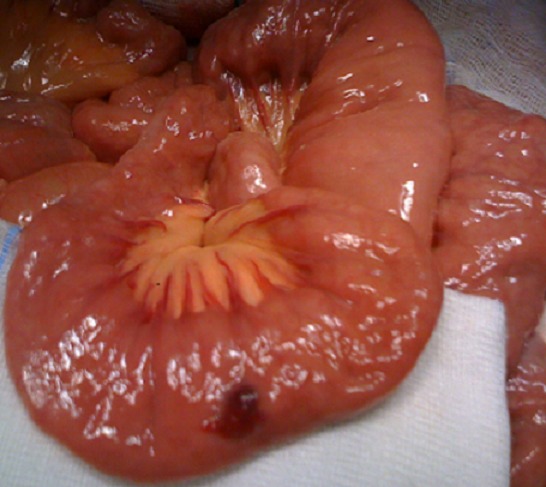
Hémangiome intestinal (après l'exérèse)

**Figure 5:**
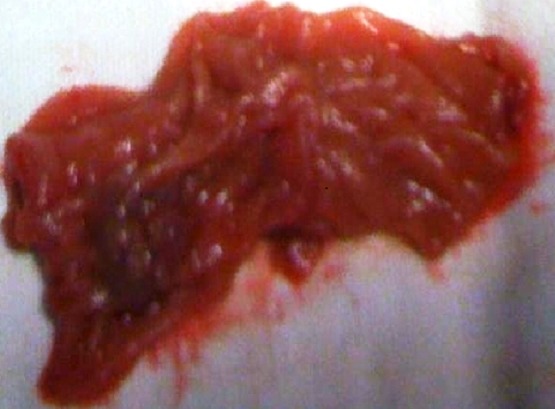
Hémangiome intestinal après l'exérèse

L'examen clinique à l'admission retrouvait un patient en mauvais état général avec une nette pâleur cutanéo muqueuse, une tension artérielle systolique à 90 mm et une diastolique à 60, un poul filant et une polypnée, le toucher rectal retrouvait des mélénas et le bilan biologique réalisé en urgence montrait une anémie hypochrome microcytaire avec un taux d'hémoglobine à 3 g dl ayant nécessité la transfusion sanguine de 6 culots globulaires, la gastroscopie réalisée après stabilisation de l’état hémodynamique ne retrouvait pas du sang dans l'estomac ni de lésions susceptibles de saigner, la coloscopie avec cathétérisme de la dernière anse iléale (DAI) était normale, devant cette hémorragie digestive extériorisée inexpliquée, il a été décidé de réaliser une VCE du grêle, cet examen a été précédé par la réalisation d'un entéroscanner qui était sans particularités (Figure 1), la VCE montrait par contre une tumeur sous muqueuse sans ulcération en surface avec stigmates de saignement récent (Figure 2, Figure 3),l'entéroscopie à double ballon n’étant pas disponible dans notre unité, le patient ayant était opéré, on retrouvait en per opératoire la tumeur de 110 cm de taille (Figure 4, Figure 5) et dont l′examen anatomopathologique confirmait la nature bénigne, l'intervention a consisté en une résection tumorale avec anastomose terminoterminale. Les suites post opératoires immédiates étaient simples. Le patient n'a pas été revu depuis dans notre unité, nous disposons d'un recul de 2 années.

## Discussion

Les angiomes sont des tumeurs vasculaires fréquemment localisées au niveau de la peau. Leur localisation digestive et notamment intestinale étant rare. L′incidence globale des hémangiomes digestifs est estimée à 1/14000 patients, et ceux de l′intestin grêle ne représentent que 3 à 4% de toutes les tumeurs de celui ci et seulement 0,3% de toutes les tumeurs du tractus gastro-intestinal [1]. Ces tumeurs sont souvent de découverte fortuite, soit lors d′une laparotomie ou à l'occasion d'une autopsie [2], elles s′observent à tout âge, avec une plus grande fréquence chez les enfants et les adolescents L’âge moyen du diagnostic étant compris entre 5 et 25 ans [3]. Les HG sont symptomatiques dans 90% des cas [4] mais leur expression clinique est très variable et non spécifique, L'occlusion intestinale par obstruction ou par invagination pourraient être des complications révélatrices, de même que la perforation ou le syndrome de malabsorption ou très rarement une séquestration plaquettaire [5], mais l'hémorragie digestive reste le mode de révélation le plus fréquent [6]. Les HG sont le plus souvent uniques, mais peuvent être multiples [7], L′aspect macroscopique habituel des HG est sessile ou polypoïde, de couleur bleutée ou parfois rouge [8]. Sur le plan histologique, Les HG peuvent être de type caverneux, capillaire ou mixte. Mais le type caverneux est le plus fréquent [5].

Le diagnostic positif des HG est souvent difficile mais indispensable pour aboutir à un traitement adéquat [9], plusieurs moyens radiologiques sont disponibles mais leur rendement diagnostique surtout en matière d'hémorragie digestive inexpliquée reste limité. Le transit du grêle classique ou par entéroclyse n'a plus de place du fait de ses des limites importantes, comme l'absence de spécificité de la majorité des lésions observées et l'incapacité de détecter les angiodysplasies, cause la plus fréquente de saignement du grêle [10]. Sa sensibilité pour la détection des tumeurs, notamment celles de petite taille, est insuffisante [11] actuellement le transit du grêle est remplacé par de nouvelles techniques radiologiques comme l′entéroscanner et l'entéro-IRM. Le scanner permet la détection des tumeurs de l'intestin grêle ainsi que les lésions extra muqueuses mais les lésions planes, les petites lésions souvent bénignes et les MAV échappent à cet examen comme dans notre cas [12].

L'entéroscanner a une sensibilité de 80 à 100%, une spécificité de 90 à 97% et une valeur prédictive négative de 95 à 100%. Les faux positifs peuvent être dus à des gros plis intestinaux (pseudo-tumeur) ou des invaginations fonctionnelles. L'intérêt de cet examen reste limité pour le diagnostic des anomalies vasculaires et des lésions muqueuses superficielles du grêle [13]. L'entéro-IRM beaucoup plus évaluée dans la maladie de Crohn a une sensibilité de l'ordre de 45 à 90% et sa spécificité est de 87 à 100% [14]. Dans le cadre du diagnostic étiologique de l'hémorragie digestive inexpliquée, l'entéroscanner est demandé pour rechercher une éventuelle lésion pouvant expliquer la carence en fer mais aussi pour éliminer un obstacle avant de mettre en place la VCE surtout chez le sujet moins de 50 ans [15] comme c'est le cas chez notre malade. L'artériographie et l'angio-scanner nécessitent une hémorragie active de haut débit pour être utiles au diagnostic, ils peuvent aussi être d'un grand intérêt thérapeutique pour emboliser les lésions hémorragiques détectées [6].

Le rendement de la radiologie en matière d'hémorragie digestive inexpliquée reste au total modeste, les explorations endoscopiques à l’ère de la VCE et de l'entéroscopie double ballon EDB sont plus en plus incontournables [16]. La capsule est un système d'imagerie sophistiqué qui permet l'exploration du tube digestif et surtout de la totalité de l'intestin grêle de façon non invasive et bien tolérée. L'exploration de l'intestin grêle par (VCE) se généralise et son rendement diagnostique est bien établi notamment chez les patients présentant un saignement digestif chronique obscur, occulte ou extériorisé comme chez le cas de notre patient. Dans le cadre des saignements digestifs obscurs, la fréquence des tumeurs intestinales, qu'elles soient malignes ou bénignes, varie entre 6,3% et 12,3% [17].

Moins de 2% des tumeurs de l'intestin grêle sont de nature maligne. Ces tumeurs malignes ont un pronostic péjoratif lié aux difficultés du diagnostic initial: 40 à 75% ont déjà métastasé au moment du diagnostic, 20 à 50% des malades ne relèvent plus de la chirurgie au moment de leur découverte [18]. La VCE pourrait modifier cette situation grâce à un diagnostic plus précoce, l'intérêt de la capsule résidant surtout dans la détection des petites tumeurs (de moins de 1 cm) qui ne peuvent être visualisées par l'entéroscanner [19] comme dans notre observation. La performance diagnostique de la vidéo capsule endoscopique varie de 55 à 81% avec une nette supériorité comparée aux examens radiologiques [20]. Dans notre observation la capsule endoscopique a permis de mettre en évidence l'hémangiome qui prenait l'aspect d'une tumeur sous muqueuse, il y avait aussi des stigmates de saignement récent au niveau de l'intestin grêle, elle a permis ainsi de poser l'indication thérapeutique et a évité au malade une éventuelle récidive hémorragique pouvant être mortelle.

La chirurgie était jusqu′à récemment le seul traitement curatif disponible des hémangiomes. La résection complète permet la guérison comme dans notre observation. Pour les petites tumeurs strictement confinées à la muqueuse, certains auteurs ont proposé la mucosectomie [3] notamment dans les localisations colorectales et gastriques [1]. En cas d′hémangiomes multiples la chirurgie à visée curative est impraticable mais l'EDB a bouleversé l′approche thérapeutique de ces lésions [18]. Certains auteurs ont eu recours à l'usage de stéroïdes, à l′embolisation ou l'administration de l′alpha-interféron, mais les résultats de ces approches médicamenteuses sont très variables [21].

## Conclusion

Les hémangiomes de l'intestin grêle sont certes des tumeurs rares, mais peuvent se manifester par des complications révélatrices, les différentes méthodes radiologiques d'exploration de l'intestin grêle sont souvent imparfaites. La vidéo capsule endoscopique examen non invasif est d'un grand apport diagnostique dans les formes hémorragiques. Le traitement des localisations greliques est essentiellement chirurgical, le pronostic est généralement bon.
